# The Vivisection Commission's Report

**Published:** 1909-06-05

**Authors:** Stephen Paget

**Affiliations:** Hon. Sec.


### Editor's Letter-Box.

### THE VIVISECTION COMMISSION'S REPORT.

*To the Editor of* The Hospital.

Sir,—The Committee of the Research Defence Society have asked me to send you a copy of a letter which has been sent to the Secretary of State for Home Affairs, as follows :—

"Research Defence Society, 70 Harley Street, W.
May 26, 1909.

To the Rt. Hon. Herbert J. Gladstone.

Sir,—We desire, as members of the Research Defence Society, to call your attention to the following facts. The Royal Commission on Experiments on Animals was appointed in 1906. It began to hear evidence in October of that year ; and during 1906 and 1907 a great amount of evidence was given by many witnesses. So long ago as December 18, 1907, the Commissioners decided that they did not wish for any further evidence ; but they met once more, on March 25, 1908, to hear evidence on a special point. Apart from this one meeting in 1908, it is now nearly a year and a half since the Commissioners ceased to hear evidence ; but they have not yet issued their report. We are of opinion that this long delay is contrary to the public interest, and is likely to prejudice the public mind. We, therefore, beg you to exercise all your influence to hasten the issue of the report."

The letter has been signed by the Earl of Cromer, President of the Society ; the Hon. Sydney Holland, Chairman of Committee ; the Duke of Abercorn, Lord Avebury, Lady Bliss, Lord Robert Cecil, Lord Cheylesmore, Sir Savile Crossley, the Hon. Walter Guinness, Sir Edwin Ray Lankester, Sir Frank Lascelles, Mr. Frederick Macmillan, the Earl of Malmesbury, Sir Patrick Manson, the Duke of Montrose, the Dean of St. Patrick's, Lord Rothschild, Mrs. Scharlieb, Professor Starling, Sir Reginald Talbot, Sir Frederick Treves, the Duke of Wellington, and the Bishop of Winchester.

I remain, your obedient servant,
Stephen Paget, Hon. Sec.

